# Expressions of TWIST1 and CD105 markers in colorectal cancer patients and their association with metastatic potential and prognosis

**DOI:** 10.1186/s13000-021-01088-1

**Published:** 2021-03-22

**Authors:** Fahimeh Fattahi, Leili Saeednejad Zanjani, Somayeh Vafaei, Zohreh Habibi Shams, Jafar Kiani, Marzieh Naseri, Elmira Gheytanchi, Zahra Madjd

**Affiliations:** 1 Oncopathology Research Center, Iran University of Medical Sciences , Postal Code: 14496-14530, Hemmat Street (Highway), Next to Milad Tower, Tehran, Iran; 2 Department of Molecular Medicine, Faculty of Advanced Technologies in Medicine, Iran University of Medical Sciences, Tehran, Iran; 3 Student Research Committee, Iran University of Medical Sciences, Tehran, Iran; 4 Department of Pathology, Iran University of Medical Sciences, Tehran, Iran

**Keywords:** TWIST1, CD105, Colorectal cancer (CRC), Immunohistochemistry (IHC), Tissue microarray (TMA)

## Abstract

**Background:**

TWIST1 and CD105, which contribute to tumor malignancy, are overexpressed in cancers. Accordingly, TWIST1 enhances epithelial-to-mesenchymal transition (EMT) and promotes the formation of cancer stem cells (CSCs). Also, CD105 is a neoangiogenesis marker in endothelial cells, which is introduced as a CSC marker in tumoral epithelial cells in several types of cancers. The present study was aimed to investigate expressions of TWIST1 and CD105 in colorectal cancer (CRC) patients.

**Methods:**

Expressions of TWIST1 and CD105 in 250 CRC tissue samples were evaluated using immunohistochemistry on tissue microarrays (TMAs). In this regard, TWIST1 expression was investigated in the subcellular locations (cytoplasm and nucleus), while CD105 was mapped in endothelial cells and cytoplasmic tumor cells of CRC tissues. The association between the expression of these markers and clinicopathological parameters, as well as survival outcomes were analyzed.

**Results:**

Results indicate a statistically significant association between higher nuclear expression levels of TWIST1 and distant metastases in CRC (*P* = 0.040) patients. In addition, it was shown that the increased nuclear expression of TWIST1 had a poor prognostic value for disease-specific survival (DSS) and progression-free survival (PFS) (*P* = 0.042, *P* = 0.043, respectively) in patients with CRC. Moreover, analysis of CD105 expression level has revealed that there is a statistically significant association between the increased expression of CD105 in tumoral epithelial cells and more advanced TNM stage (*P* = 0.050).

**Conclusions:**

Our results demonstrate that nuclear TWIST1 and cytoplasmic CD105 expressions in tumor cells had associations with more aggressive tumor behavior and more advanced diseases in CRC cases.

## Background

Colorectal cancer (CRC), as a major health concern in both genders, is known as the second leading cause of cancer death worldwide. CRC ranks as the third incidence rate with over 1.8 million new cases reported by the World Health Organization in 2018 [1]. Despite the advances in screening tests and treatment of CRC, the 5-year survival rate was estimated as 65% in high-income countries; however, it has remained less than 50% in low-income developing countries [2]. Nowadays, there is a need for discovering universal biomarkers in CRC to be used in clinical practice; therefore, performing studies on CRC biomarkers is increasing. The findings of such studies may affect the diagnosis and prognosis of patients with CRC, which may help in increasing their lifespans [2, 3].

Changes in genetic and epigenetic due to some factors can lead to epithelial-to-mesenchymal transition (EMT) in epithelial neoplastic cells [4], which causes migration of these cells from primary tissue to stromal components [5]. Epithelial cells lose intercellular junctions, reorganize cell cytoskeleton, and gain migratory properties during the EMT process, which all increase mobility of cells [6]. Several studies have shown that EMT is importantly associated with a poor prognosis in the cancerous patients [7]. In this regard, several markers to induce EMT have been described such as TWIST proteins, matrix metallopeptidases (MMPs), transforming growth factor beta (TGF-β), and TGF-β receptor [6, 8].

Most of the tumor cells use EMT-associated transcription factors (EMT-TFs), which were identified as key participants in the EMT program [9]. TWIST1, as one of the EMT-TFs, is involved in EMT through the downregulation of E-cadherin and regulates apoptosis by interacting with p53 protein [10, 11]. Accordingly, this protein plays multiple roles in cancers and mediates cell migration and differentiation. In addition, some reports indicated that TWIST1 is associated with angiogenesis and stemness in various cancers [11–13]. As described earlier in a review report, overexpression and role of TWIST1 were observed in various cancers such as prostatic cancer, gastric cancer, breast cancer, and CRC [11]. Findings indicated that CRC patients with positive TWIST1 expression in tumor cells have low survival rates [14]. In addition, some evidence showed that TWIST1 may regulate endothelial markers, so that overexpression of TWIST1 in oral squamous carcinoma cells is associated with the expression and activation of CD105 marker [15, 16].

TGF-β receptor is an important key inducer of EMT-TFs, which changes expression levels of EMT markers in CRC [17, 18]. CD105 (endoglin), as a neoangiogenesis-related protein, is known as a component of TGF-β receptor and a regulator for TGF-β signaling [19]. Studies performed on cancer have reported that CD105 may induce EMT program through EMT-TFs and play a functional role in maintaining cancer stem cells (CSCs) [20, 21]. Moreover, CD105 is considered as an appropriate marker for angiogenesis that regulates proliferating endothelial cells and blood vessels’ development [22]. CD105 contributes to all malignancies, which can be up-regulated by hypoxia and TGF-βs; therefore, it can help in promoting tumor proliferation and metastasis in several types of cancer [22–24]. Studies have shown that overexpression of endothelial CD105 is associated with the advanced cancer, so CD105 can be considered as a prognosis marker and as a targeted therapy [25–27]. Also, the increased expression of CD105 was observed in aggressive and metastatic CRC patients [14, 28].

Although TWIST1 and CD105 expressions have been shown as poor prognosis markers in CRC, very little information has been reported on the expression levels of these markers based on subcellular location in CRC samples. Thus, in this study, we investigated the association between cytoplasmic and nuclear TWIST1 expression, as well as the association of CD105 expression in endothelial cells and cytoplasmic tumor cells with clinicopathological parameters and survival outcomes in patients with CRC through immunohistochemistry (IHC) staining using tissue microarray (TMA) method.

## Methods

### Patients and tumor samples collection

In the present study, formalin-fixed paraffin-embedded (FFPE) blocks of 250 CRC patients were collected from the labs of three referral hospitals (Hashemi Nejad, Firoozgar, and Rasoole Akram) in Tehran, Iran, from 2012 to 2018. Patients who had undergone surgical treatment by receiving no relevant chemotherapy or radiotherapy were included in this study. The hematoxylin and eosin (H & E) stained slides and the information of clinicopathological parameters including age, gender, tumor size, tumor differentiation, TNM stage, vascular invasion (VI), lymph node invasion (LNI), neural invasion (NI), distant metastasis, and tumor recurrence were also collected. Moreover, 50 adjacent normal tissues were included in this study. The enrolled patients were followed up until March 2020 and patient outcome information including disease-specific survival (DSS), which was defined as the time from tumor resection to death time due to CRC and progression-free survival (PFS), which is the time from tumor resection until last follow-up (patients with no evidence of disease, recurrence or progression), were collected. In addition, pathological stage was described in terms of cancer staging manual of the American Joint Committee on Cancer (AJCC) in 2018 [29].

### Tissue microarray (TMA) construction

TMA blocks were constructed as described earlier [30]. Three representative tumor region cores were microscopically selected on H & E slides. The representative areas for TMA blocks included both superficial epithelial foci and deep foci (where the tumor invades mesenchymal tissue). Correspondingly, three cores were randomly extracted from representative areas using a 0.6-mm punch of FFPE tissue blocks from CRC patients. The cores were placed using a precision arraying instrument (Tissue Arrayer Minicore; ALPHELYS, Plaisir, France) into recipient TMA blocks. Five-micrometer-thick were obtained by cutting array blocks and putting them on adhesive slides. TMA blocks were built in three copies for each specimen of different areas and the mean scoring of the three cores was then calculated as the final score. It was shown that three copies in TMA-IHC method could increase accuracy up to 99%, while the single core has 90% accuracy [31].

### Immunohistochemistry (IHC) staining

IHC staining was performed for TWIST1 and CD105 on those sections that were cut from the TMA blocks. TMA sections were deparaffinized (for 30 min at 60 °C), rehydrated with xylenes, and then graded by ethanol. Afterward, they were incubated in 3% H2O2 for 20 min at room temperature until endogenous peroxidase blockage on sections. Thereafter, antigen tissue was exposed by antigen retrieval processing that includes heating tissue sections in sodium citrate buffer (pH = 6.0) for TWIST1 and Tris-EDTA (pH = 9.0) for CD105 by autoclave for 10 min. Subsequently, the primary antibodies were separately incubated overnight at 4 °C with rabbit monoclonal antibody against TWIST1 and CD105 (TWIST1: ab49254; 1:80 dilution and CD105: ab169545; 1:400 dilution, Abcam, Cambridge, MA, USA). After washing for 3 times in Tris-buffered saline, the secondary antibody was incubated for tissue slides with anti-rabbit/anti-mouse Envision (Dako, Glostrup, Denmark) for 30 min. In order to visualize the antigen, 3, 3′-diaminobenzidine substrate (DAB) (Dako, Glostrup, Denmark) was applied as chromogen for 10 min at room temperature. The slides were then treated with hematoxylin (Dako, Glostrup, Denmark), as a counterstain, for 3 min. Finally, to prepare the scoring, the slides were dehydrated in alcohol and then cleared in xylenes. Positive and negative controls were used in each run of the experiment. Manufacturer’s recommendations for positive control tissue were human normal testis and human normal kidney tissues for TWIST1 and CD105 staining, respectively. For negative controls, primary antibodies were replaced by Tris-buffered saline (TBS).

### Assessment of immunohistochemical staining

Immunohistochemical markers were also assessed by two pathologists (Z.HS and Z.M) who were blinded to pathological parameters and the patients҆ outcomes. Initially, 10 X magnification was used for TMA slides to obtain a general impression of distribution cells, and positive cells were then investigated by applying a semi-quantitative scoring system at higher magnifications (40 X). A consensus was reached by two pathologists for scoring samples. Also, staining was determined by the immunostaining intensity (0: absent, 1: weak, 2: moderate or 3: strong) and the percentage of positive tumor cells was also categorized as follows: < 25, 25 to 50%, 51 to 75%, and > 75%. Finally, H-score was assigned based on multiplying the intensity score by the percentage of the stained cells, which was given to each core ranging from 0 to 300 [32]. The average of the three cores was calculated as the final score. In this study, mean H-score was chosen to categories’ samples as high or low expression.

### Statistical analysis

Statistical analysis of data was performed by SPSS v.22.0 software (IBM Corp., Armonk, NY, USA). Kruskal–Wallis and Mann–Whitney *U* tests were then performed to compare groups. Chi-square and Spearman’s correlation tests were also used to analyze the significance of association and correlation between expression level of the markers and clinicopathological parameters.

The survival analysis was performed using Kaplan–Meier method, and Log-rank test was then applied to compare the estimated curves between the groups (high expression compared to low expression). Univariate and multivariate analyses were performed by Cox proportional hazard models. In order to determine which one of the variables affected DSS or PFS, those variables that significantly affected the survival in univariate analysis were used in multivariable analyses. Quantitative data were reported as mean (SD) and median (Q1, Q3). *P*-values less than 0.05 (*P* ≤ 0.05) were considered as statistically significant.

## Results

### Patients’ characteristics

In the current study, out of 250 CRC cases in total, 223 patients including 114 men (51.1%) and 109 women (48.9%) were evaluated for TWIST1 expression. Patients’ ages ranged from 20 to 91 years old (mean age = 59, SD = 14.6). Whereas, 208 CRC patients including 111 men (53.4%) and 97 women (46.6%) with the age range of 25–88 years old (mean age = 59, SD = 13.6) were investigated for CD105 expression. The clinicopathological features for our samples are shown in Table 1.

**Table 1:** Patients and tumor pathological parameters of the studied population

Patients and tumor characteristics	TWIST1 marker	CD105 marker
Number of CRC samples	223	208
Mean age, years (Range)	59 (20–91)	59 (25–88)
≤ Mean age	110 (49.3)	105 (50.5)
> Mean age	113 (50.7)	103 (49.5)
Gender		
Male	114 (51.1)	111 (53.4)
Female	109 (48.9)	97 (46.6)
(Male/Female)	1.04	1.14
Mean tumor size (cm)	5	5
≤ Mean	156 (70.0)	145 (69.7)
> Mean	67 (30.0)	63 (30.3)
Tumor differentiation		
Well	103 (46.2)	89 (42.8)
Moderate	104 (46.6)	107 (51.4)
Poor	16 (7.2)	12 (5.8)
TNM stage		
I	40 (17.9)	29 (13.9)
II	98 (43.9)	92 (44.2)
III	79 (35.4)	80 (38.5)
IV	6 (2.7)	7 (3.4)
Vascular invasion (VI)		
Present	36 (16.1)	37 (17.8)
Absent	187 (83.9)	171 (82.2)
Lymph node invasion (LNI)		
Involved	82 (36.8)	83 (39.9)
None	141 (63.2)	125 (60.1)
Neural invasion (NI)		
Involved	47 (21.1)	57 (27.4)
None	176 (78.9)	151 (72.6)
Distant metastasis		
Present	37 (16.6)	30 (14.4)
Absent	100 (44.8)	90 (43.3)
Not identified	86 (38.6)	88 (42.3)
Tumor recurrence		
Yes	42 (18.8)	36 (17.3)
No	95 (42.6)	85 (40.9)
Not identified	86 (38.6)	87 (41.8)

### Expression of TWIST1 and CD105 markers in CRC patients

**Fig. 1:**
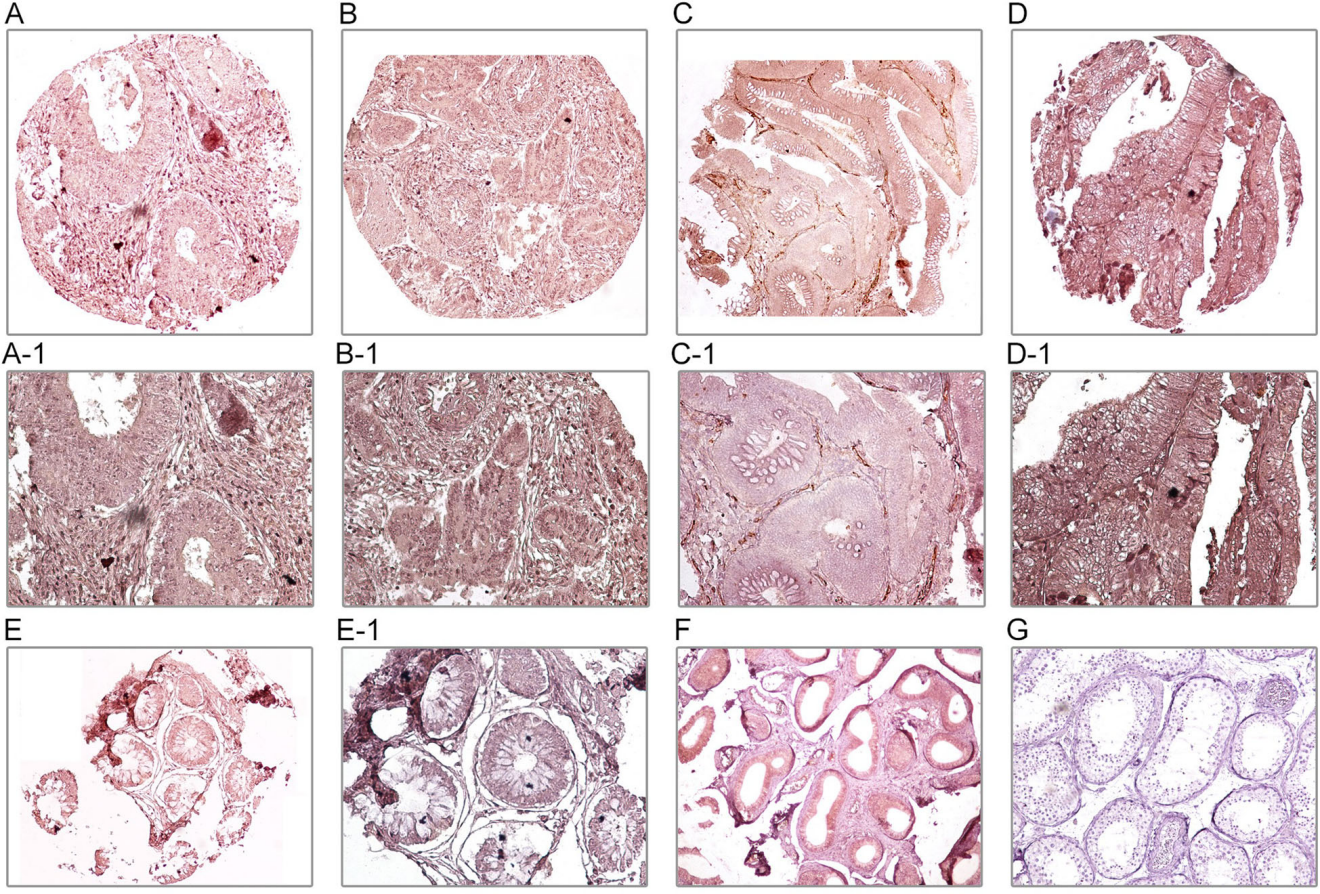
Immunohistochimecal staining of TWIST1 in colorectal cancer (CRC) tissues, adjacent normal tissues, and normal testis controls. Nuclear TWIST1 expression in tumor cells: low expression **a-a1** and high expression **b-b1**. Cytoplasmic TWIST1 expression in tumor cells: low expression **c-c1** and high expression **d-d1**. Adjacent normal tissues **e-e1**. Normal testis tissues as controls: positive **f** and negative **g**

**Fig. 2:**
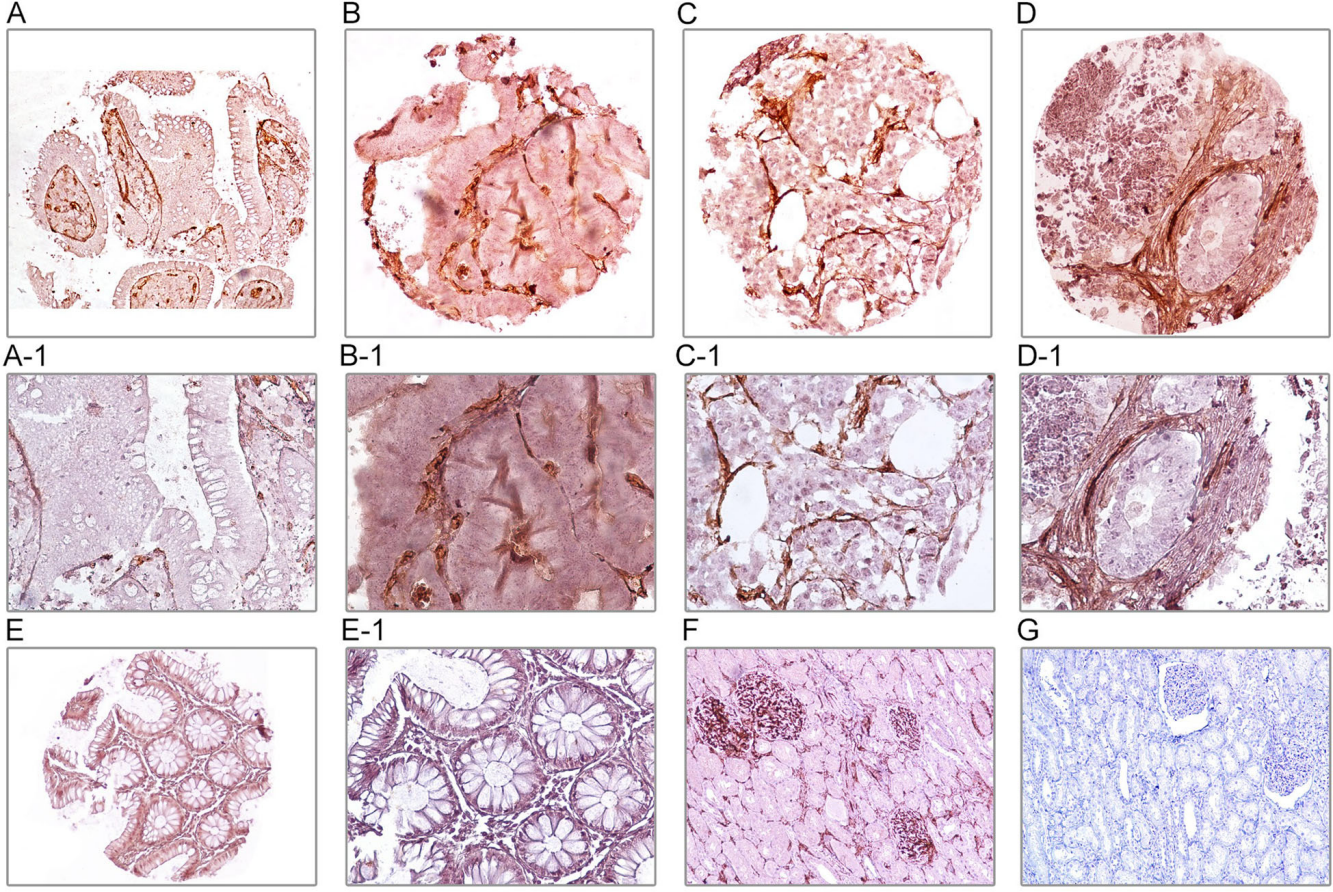
Immunohistochimecal staining of CD105 in colorectal cancer tissues (CRC), adjacent normal tissues, and normal testis controls. Cytoplasmic CD105 expression in tumor cells: low expression **a-a1** and high expression **b-b1**. CD105 expression in endothelial cells: low expression **c-c1** and high expression **d-d1**. Adjacent normal tissues **e-e1**. Normal kidney tissues as controls: positive **f** and negative **g**

The expression levels of TWIST1 and CD105 markers were investigated using IHC on TMAs of CRC patients. Scoring was done in terms of three methods, which include the following: intensity of staining, percentage of positive cells, and H-score (Table 2). TWIST1 was expressed at different sites (both nucleus and cytoplasm) in the tumor cells, while CD105 was detected in endothelial cells and cytoplasm of tumor cells of the obtained CRC samples. Lower levels of expressions of TWIST1 and CD105 were observed in adjacent normal tissues compared to the tumor samples. In human normal testis sample, as a positive control for TWIST1, presented cytoplasmic staining in seminiferous epithelial cells. Moreover, human normal kidney tissue that was used as a positive control for CD105 marker showed staining in all endothelial cells, tubule capillaries, and glomerular endothelial cells, but no staining was shown in tubule cells (Figs. 1 and 2).

**Table 2:** TWIST1 and CD105 expression (Intensity of staining, percentage of positive tumor cells, and H-score) in colorectal cancer (CRC) samples

Scoring system	Cytoplasmic TWIST1 *N* (%)	Nuclear TWIST1 N (%)	Cytoplasmic CD105 *N* (%)	Endothelial CD105 N (%)
Intensity of staining				
No staining (0)	0 (0.0)	0 (0.0)	44 (21.2)	0 (0.0)
Weak (+ 1)	111 (49.8)	69 (30.9)	161 (77.4)	0 (0.0)
Moderate (+ 2)	106 (47.5)	123 (55.2)	3 (1.4)	25 (12.0)
Strong (+ 3)	6 (2.7)	31 (13.9)	0 (0.0)	183 (88.0)
Percentage of positive tumor cells				
< 25%	0 (0.0)	0 (0.0)	121 (58.2)	0 (0.0)
25–50%	0 (0.0)	1 (0.4)	60 (28.8)	0 (0.0)
51–75%	3 (1.3)	20 (9.0)	13 (6.3)	6 (2.9)
> 75%	220 (98.7)	202 (90.6)	14 (6.7)	202 (97.1)
H-score cut off	149	172	26	284
Low	113 (50.7)	87 (39.0)	121 (58.2)	34 (16.3)
High	110 (49.3)	136 (61.0)	87 (41.8)	174 (83.7)
Total	223	223	208	208

H-score indicates histological score

### Association between cytoplasmic and nuclear expression of TWIST1 with clinicopathological parameters

TWIST1 staining was observed in cytoplasm and nucleus of tumor cells in all the obtained CRC samples. Chi-square test analysis showed a statistically significant association between the increased expression of nuclear TWIST1 expression and distant metastasis (*P* = 0.040). Whereas, no statistically significant association was observed between cytoplasmic TWIST1 expression levels and clinicopathological parameters in the cases studied (Table 3).

**Table 3:** The association between cytoplasmic and nuclear TWIST1 expressions and clinicopathological parameters of colorectal cancer (CRC) samples (H-score) (*P* value; Pearson’s χ2 test)

Patients and tumor characteristics	Total samples *N* (%)	H-score (cut off = 149) *N* (%) Cytoplasmic TWIST1 expression		*P*-value	H-score (cut off = 172) *N* (%) Nuclear TWIST1 expression		*P*-value
		Low (≤ 149)	High (> 149)		Low (≤ 172)	High (> 172)	
Number of CRC samples	223	113 (50.7)	110 (49.3)		87 (39.0)	136 (61.0)	
Mean age, years (Range)	59 (20–91)			0.159			0.262
≤ Mean age	110 (49.3)	61 (54.0)	49 (44.5)		47 (54.0)	63 (46.3)	
> Mean age	113 (50.7)	52 (46.0)	61 (55.5)		40 (46.0)	73 (53.7)	
Gender				0.095			0.129
Male	114 (51.1)	64 (56.6)	50 (45.5)		50 (57.5)	64 (47.1)	
Female	109 (48.9)	49 (43.4)	60 (54.5)		37 (42.5)	72 (52.9)	
(Male/Female)	1.04	1.30	0.83		1.35	0.88	
Mean tumor size (cm)	5			0.389			0.522
≤ Mean	156 (70.0)	82 (72.6)	74 (67.3)		63 (72.4)	93 (68.4)	
> Mean	67 (30.0)	31 (27.4)	36 (32.7)		24 (27.6)	43 (31.6)	
Tumor differentiation				0.549			0.405
Well	103 (46.2)	53 (46.9)	50 (45.5)		45 (51.7)	58 (42.6)	
Moderate	104 (46.6)	50 (44.2)	54 (49.1)		36 (41.4)	68 (50.0)	
Poor	16 (7.2)	10 (8.8)	6 (5.5)		6 (6.9)	10 (7.4)	
TNM stage				0.932			0.831
I	40 (17.9)	19 (16.8)	21 (19.1)		16 (18.4)	24 (17.6)	
II	98 (43.9)	52 (46.0)	46 (41.8)		40 (46.0)	58 (42.6)	
III	79 (35.4)	39 (34.5)	40 (36.4)		28 (32.2)	51 (37.5)	
IV	6 (2.7)	3 (2.7)	3 (2.7)		3 (3.4)	3 (2.2)	
Vascular invasion (VI)				0.783			0.445
Present	36 (16.1)	19 (16.8)	17 (15.5)		12 (13.8)	24 (17.6)	
Absent	187 (83.9)	94 (83.2)	93 (84.5)		75 (86.2)	112 (82.4)	
Lymph node invasion (LNI)				0.878			0.571
Involved	82 (36.8)	41 (36.3)	41 (37.3)		30 (34.5)	52 (38.2)	
None	141 (63.2)	72 (63.7)	69 (62.7)		57 (65.5)	84 (61.8)	
Neural invasion (NI)				0.697			0.653
Involved	47 (21.1)	25 (22.1)	22 (20.0)		17 (19.5)	30 (22.1)	
None	176 (78.9)	88 (77.9)	88 (80.0)		70 (80.5)	106 (77.9)	
Distant metastasis				0.234			***0.045***
Present	37 (16.6)	15 (13.3)	22 (20.0)		8 (9.2)	29 (21.3)	
Absent	100 (44.8)	52 (46.0)	48 (43.6)		40 (46.0)	60 (44.1)	
Not identified	86 (38.6)	46 (40.7)	40 (36.4)		39 (44.8)	47 (34.6)	
Tumor recurrence				0.568			0.149
Yes	42 (18.8)	19 (16.8)	23 (20.9)		11 (12.7)	31 (22.8)	
No	95 (42.6)	48 (42.5)	47 (42.7)		37 (42.5)	58 (42.6)	
Not identified	86 (38.6)	46 (40.7)	40 (36.4)		39 (44.8)	47 (34.6)	

H-score indicates histological score

Values in bold are statistically significant

### Association between tumoral cytoplasmic and endothelial expressions of CD105 with clinicopathological parameters

Endothelial CD105 expression was observed in all the samples of CRC, while cytoplasmic expression of CD105 in tumor cells was detected in 164 (78.8%) CRC samples. Moreover, the results of Spearman’s correlation test demonstrated a statistically significant positive correlation between CD105 expression in endothelial and tumor cells of CRC samples (*P* = 0.007, r_s_: 0.18).

**Fig. 3:**
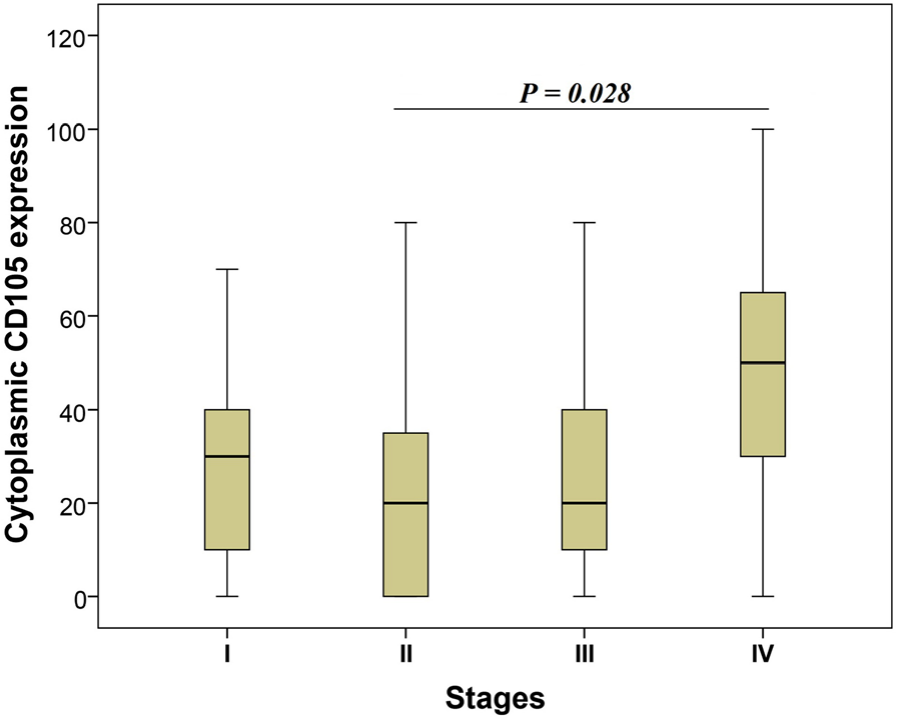
Box plot of cytoplasmic CD105 expression levels for TNM stage in colorectal cancer. Based on the standard definitions, each box-plot shows the median (bold line) and interquartile lines (box). The results of Mann–Whitney *U* test showed that there was a statistically significant difference in terms of the mean expression levels of CD105 between stage II and IV in tumor cells (*P* = 0.028)

The results of Chi-square test indicated a statistically significant association between cytoplasmic CD105 expression levels and the advanced TNM stage (*P* = 0.050). Also, Kruskal–Wallis test to compare the mean expression levels of CD105 in different groups, revealed a statistically significant difference between cytoplasmic CD105 expression and various TNM stages (I–IV) (P = 0.050). Furthermore, Mann–Whitney *U* test demonstrated a significant difference in terms of the mean level of cytoplasmic CD105 expression between TNM stages II and IV (*P* = 0.028) (Fig. 3). The mean expression levels of cytoplasmic CD105 expression in tumor cells for stages II and IV were 22 and 48, respectively. No significant association was observed between cytoplasmic CD105 expression levels and the other clinicopathological parameters in CRC cases (Table 4)**.**

**Table 4:** The association between cytoplasmic and endothelial CD105 expressions and clinicopathological parameters of colorectal cancer (CRC) samples (H-score) (*P* value; Pearson’s χ2 test)

Patients and tumor characteristics	Total samples *N* (%)	H-score (cut off = 26) *N* (%) Cytoplasmic CD105 expression		*P*-value	H-score (cut off = 284) *N* (%) Endothelial CD105 expression		*P*-value
		Low (≤ 26)	High (> 26)		Low (≤ 284)	High (> 284)	
Number of CRC samples	208	121 (58.2)	87 (41.8)		34 (16.3)	174 (83.7)	
Mean age, years (Range)	59 (25–88)			0.796			0.358
≤ Mean age	105 (50.5)	62 (51.2)	43 (49.4)		20 (58.9)	85 (48.9)	
> Mean age	103 (49.5)	59 (48.8)	44 (50.6)		14 (41.1)	89 (51.1)	
Gender				0.872			0.305
Male	111 (53.4)	64 (52.9)	47 (54.0)		15 (44.1)	96 (55.2)	
Female	97 (46.6)	57 (47.1)	40 (46.0)		19 (55.9)	78 (44.8)	
(Male/Female)	1.14	1.12	1.17		0.78	1.23	
Mean tumor size (cm)	5			0.472			0.693
≤ Mean	145 (69.7)	82 (67.8)	63 (72.4)		23 (67.7)	122 (70.1)	
> Mean	63 (30.3)	39 (32.2)	24 (27.6)		11 (32.3)	52 (29.9)	
Tumor differentiation				0.605			0.450
Well	89 (42.8)	55 (45.5)	34 (39.1)		12 (35.2)	77 (44.3)	
Moderate	107 (51.4)	60 (49.6)	47 (54.0)		21 (61.7)	86 (49.4)	
Poor	12 (5.8)	6 (5.0)	6 (6.9)		1 (3.1)	11 (6.3)	
TNM stage				***0.050***			0.631
I	29 (13.9)	14 (11.6)	15 (17.2)		7 (20.5)	22 (12.6)	
П	92 (44.2)	62 (51.2)	30 (34.5)		13 (38.2)	79 (45.4)	
III	80 (38.5)	43 (35.5)	37 (42.5)		13 (38.2)	67 (38.5)	
IV	7 (3.4)	2 (1.7)	5 (5.7)		1 (3.1)	6 (3.4)	
Vascular invasion (VI)				0.587			0.711
Present	37 (17.8)	23 (19.0)	14 (16.1)		6 (17.6)	31 (17.8)	
Absent	171 (82.2)	98 (81.0)	73 (83.9)		28 (82.4)	143 (82.2)	
Lymph node invasion (LNI)				0.713			0.978
Involved	83 (39.9)	47 (38.8)	36 (41.4)		14 (41.1)	69 (39.7)	
None	125 (60.1)	74 (61.2)	51 (58.6)		20 (58.9)	105 (60.3)	
Neural invasion (NI)				0.371			0.241
Involved	57 (27.4)	36 (29.8)	21 (24.1)		12 (35.2)	45 (25.9)	
None	151 (72.6)	85 (70.2)	66 (75.9)		22 (64.8)	129 (74.1)	
Distant metastasis				0.662			0.438
Present	30 (14.5)	20 (16.5)	10 (11.5)		3 (8.9)	27 (15.5)	
Absent	91 (43.7)	57 (47.1)	34 (39.1)		16 (47.0)	75 (43.1)	
Not identified	87 (41.8)	44 (36.4)	43 (49.4)		15 (44.1)	72 (41.4)	
Tumor recurrence				0.707			0.530
Yes	36 (17.3)	22 (18.1)	14 (16.1)		4 (11.8)	32 (18.4)	
No	85 (40.9)	55 (45.5)	30 (34.5)		14 (41.2)	71 (40.8)	
Not identified	87 (41.8)	44 (36.4)	43 (49.4)		16 (47.0)	71 (40.8)	

H-score indicates histological score

Values in bold are statistically significant

### Prognostic value of TWIST1 expression for clinical outcome

Of 223 patients included in this study, follow-up data were available only for 138 CRC samples. Also, the follow-up period was between 1 and 105 months with mean and median of follow-up time as 38 (SD = 23.3) and 38 (Q1 = 20, Q3 = 53) months, respectively. Ninety-three (67.9%) cases had no history of recurrence and distant metastasis, while 44 (32.1%) of them reported a history of these events’ occurrence. Distant metastasis and recurrence were observed in 37 (27.0%) and 42 (30.7%) patients, whereas 100 (73.0%) and 95 (69.3%) patients were negative, respectively. During the follow-up period, cancer-related death was seen in 43 (31.1%) patients.

### Survival outcomes based on expression of TWIST1

**Fig. 4:**
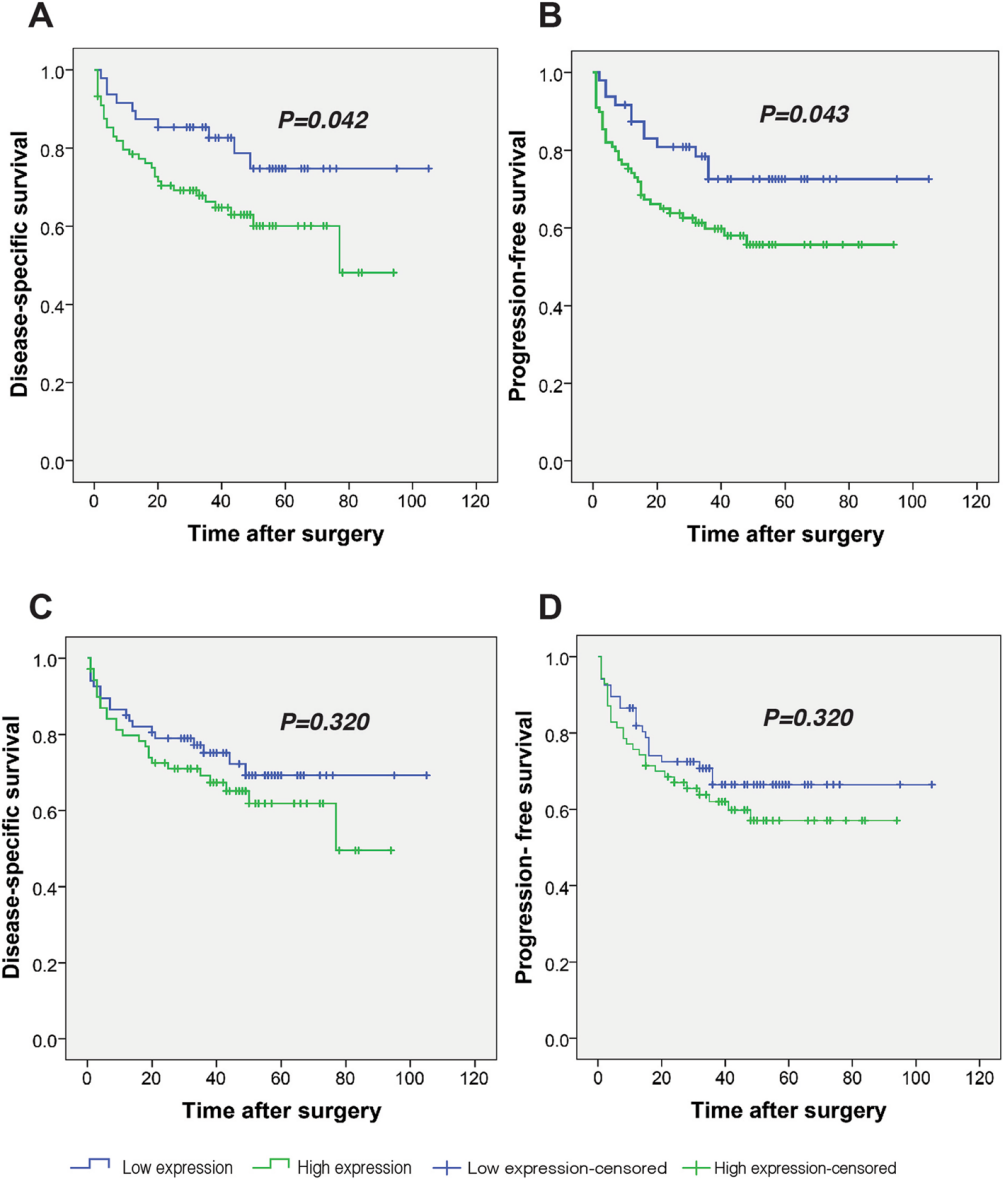
Kaplan-Meier curves for disease-specific survival (DSS) and progression-free survival (PFS) according to the expression levels of TWIST1 in colorectal cancer. (Nuclear and cytoplasmic TWIST1 expression in tumor cells was divided into two groups as follows: high and low expression levels). **a** Log Rank test showed that patients with a high nuclear TWIST1 expression had shorter DSS and **b** PFS compared to patients with a low nuclear TWIST1 expression (*P* = 0.042 and *P* = 0.043, respectively). **c, d** The Kaplan–Meier survival analysis showed that there were no significant differences in terms of survival (DSS and PFS) between patients with high and low cytoplasmic TWIST1 expressions (Survival analysis: DSS (*P* = 0.320) and PFS (*P* = 0.320))

The results of Kaplan-Meier analysis demonstrated a statistically significant differences in DSS between the patients with high and low expression levels of nuclear TWIST1 (Log Rank test, *P* = 0.042) (Fig. 4a). The mean DSS time was 60 (SD = 4.5) months for patients with high expression levels of nuclear TWIST1, and 84 (SD = 5.7) months for patients with low expression levels of that. The 5-year DSS survival rates of the patients who expressed high and low nuclear expressions of TWIST1 were 60 and 74%, respectively (*P* = 0.030). Moreover, the results of survival analysis indicated that patients with high expression levels of nuclear TWIST1 had significantly lower PFS (Log Rank test, *P* = 0.043) (Fig. 4b). In this regard, mean PFS time for patients with high nuclear TWIST1 expression was calculated as 58 (SD = 4.4) months, while it was 81 (SD = 5.9) months for patients with low nuclear TWIST1 expression. The 5-year PFS survival rates of the patients who expressed high and low nuclear expressions of TWIST1 were 55 and 72%, respectively (*P* = 0.026). Kaplan-Meier analysis showed no significant difference in survival analyses (DSS and PFS) between the patients with high and low cytoplasmic expressions’ levels of TWIST1 (Log Rank test, DSS and PFS analysis for cytoplasmic TWIS1 expression, *P* = 0.320 and P = 0.320, respectively) (Fig. 4c and d).

Also, univariate and multivariate analyses were performed to investigate whether TWIST1 expression is an independent prognostic factor of DSS or PFS, as well as assessing the clinical significance of various parameters that might affect survival outcomes among patients with CRC. Univariate analysis has demonstrated that several parameters including nuclear TWIST1 expression (*P* = 0.048), tumor size (*P* = 0.040), tumor differentiation (*P* <  0.001), vascular invasion (*P* = 0.030), neural invasion (*P* <  0.001), lymph node invasion (*P* = 0.033), distant metastasis (*P* <  0.001), and tumor recurrence (*P* <  0.001) may affect DSS (Table 5). Accordingly, the results of univariate analysis for PFS including nuclear TWIST1 expression (*P* = 0.049), tumor differentiation (*P* <  0.001), neural invasion (*P* <  0.001), lymph node invasion (*P* = 0.038), distant metastasis (*P* <  0.001), and tumor recurrence (*P* <  0.001), are summarized in Table 6. While multivariate analysis indicated that only tumor differentiation is an independent prognostic factor that affect the DSS and PFS of patients with CRC (Cox regression, DSS and PFS analysis, *P* = 0.003 and *P* <  0.001, respectively).

**Table 5:** Univariate and multivariate Cox regression analyses of potential prognostic factor for disease-specific survival (DSS) in patients with colorectal cancer

Covariate	Univariate analysis		Multivariate analysis	
	HR (95% CI)	*P*-value	HR (95% CI)	*P*-value
Nuclear TWIST1 expression High versus Low	2.04 (1.0–4.1)	**0.048**	1.6 (0.8–3.4)	0.158
Tumor size (cm)	1.8 (1.0–3.5)	**0.040**	1.5 (0.8–2.8)	0.168
Tumor differentiation		**< 0.001**		**0.003**
Moderate versus well	2.7 (1.3–5.5)	**0.004**	2.4 (1.2–4.9)	**0.013**
Poor versus well	6.1 (2.4–15.7)	**< 0.001**	5.0 (1.9–13.0)	**0.001**
Vascular invasion (VI)	0.4 (0.2–0.9)	**0.030**	–	–
Neural invasion (NI)	0.3 (0.1–0.5)	**< 0.001**	–	–
Lymph node invasion (LNI)	0.5 (0.2–0.9)	**0.033**	–	–
Distant metastasis	0.1 (0.0–0.2)	**< 0.001**	–	–
Tumor recurrence	0.1)0.05–0.20)	**< 0.001**	–	–

H-score indicates histological score

Values in bold are statistically significant

The variables with *P* value less than 0.05 were included in multivariable analyses

*HR* hazard ratio, *CI* confidence interval

**Table 6:** Univariate and multivariate Cox regression analyses of potential prognostic factor for progression- free survival (PFS) in patients with colorectal cancer

Covariate	Univariate analysis		Multivariate analysis	
	HR (95% CI)	***P-value***	HR (95% CI)	***P-value***
Nuclear TWIST1 expression High versus Low	1.9 (1.0–3.6)	**0.049**	1.62 (0.84–3.13)	0.149
Tumor differentiation		**< 0.001**		**< 0.001**
Moderate versus well	2.5 (1.3–4.7)	**0.005**	2.2 (1.1–4.3)	**0.013**
Poor versus well	6.3 (2.6–15.0)	**< 0.001**	5.4 (2.2–13.3)	**< 0.001**
Neural invasion (NI)	0.3 (0.1–0.5)	**< 0.001**	–	–
Lymph node invasion (LNI)	0.5 (0.3–0.9)	**0.038**	–	–
Distant metastasis	0.1 (0.0–0.3)	**< 0.001**	–	–
Tumor recurrence	0.1 (0.0–0.2)	**< 0.001**	–	–

H-score indicates histological score

Values in bold are statistically significant

The variables with *P* value less than 0.05 were included in multivariable analyses

hazard ratio, *CI* confidence interval

### Prognostic value of CD105 expression for clinical outcome

Follow-up data of 121 CRC samples were available for CD105 expression analysis. The range of follow up time was from 1 to 105 months with the mean follow-up time of 37 (SD = 25.1) and median of 35 months (Q1 = 20, Q3 = 50). Eighty-three (68.8%) patients had no history of recurrence and distant metastasis, while in 38 (31.2%) patients reported history of these events’ occurrence. Distant metastasis and recurrence were observed in 30 (24.7%) and 36 (29.8%) patients, whereas 91 (75.3%) and 85 (70.2%) patients were negative, respectively. During the follow-up period, cancer-related death has occurred in 37 (30.5%) patients.

### Survival outcomes based on expression of CD105

**Fig. 5:**
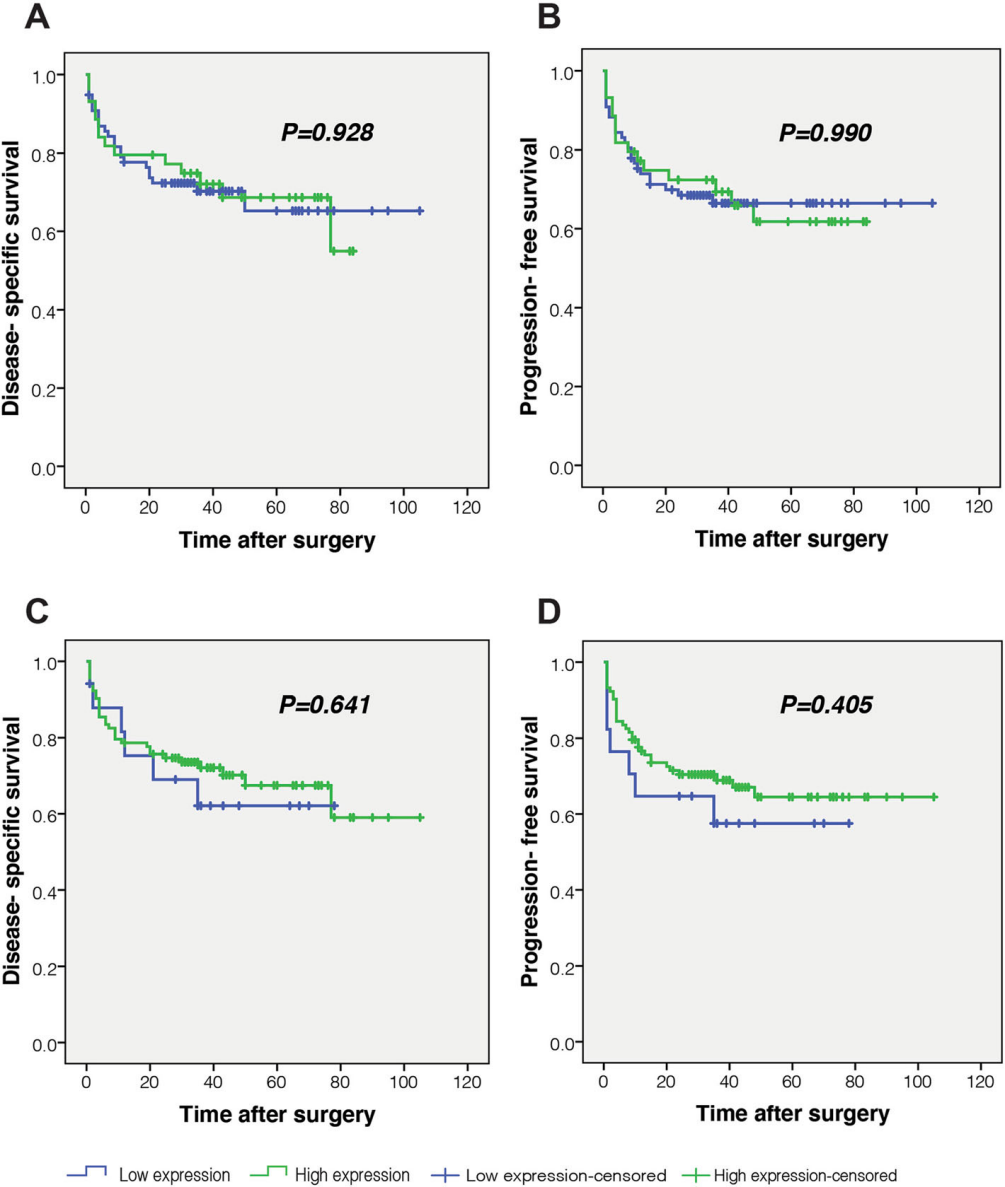
Kaplan-Meier curves for disease-specific survival (DSS) and (PFS) according to the expression levels of CD105 in colorectal cancer. (Endothelial and cytoplasmic CD105 expression in tumor cells was divided into two groups: high and low expressions’ levels). **a, b** Log Rank test showed no significant differences in survival (DSS and PFS) between patients with high and low cytoplasmic CD105 expressions in tumor cells (Survival analysis: DSS (*P* = 0.928) and PFS (*P* = 0.990)). **c, d** There were no significant differences in terms of survival (DSS and PFS) between patients with high and low CD105 expressions in endothelial cells using Log Rank test (Survival analysis: DSS (*P* = 0.641) and PFS (*P* = 0.405))

The results of Kaplan-Meier curves showed that there were no significant differences in terms of the survival analysis of DSS or PFS between the patients with high and low expression’ levels of CD105 (Log Rank test, DSS and PFS analysis for cytoplasmic CD105 expression in tumor cells, *P* = 0.928 and *P* = 0.990, respectively) (Fig. 5 A and 5B) and (Log Rank test, DSS and PFS analysis for endothelial CD105 expression, *P* = 0.641 and *P* = 0.405, respectively) (Fig. 5c and d).

In addition, the results of univariate and multivariate analyses showed that the clinicopathological parameters cannot be considered as significant factors for the DSS or PFS of patients with CRC.

## Discussion

EMT is an important biological concept of cell plasticity that contributes to the progression of cancer. EMT process is associated with some changes in motility and invasion ability of cells. Moreover, EMT is initiated with some alterations in signaling pathway’s regulation and EMT-TFs expression such as TGF-β signaling and TWIST1 [6]. Some evidence indicated that the alteration in the EMT-TFs activation is due to changes in signaling cascades, which may affect clinical outcomes related to cancer. Therefore, the signaling pathways leading to these changes can be used as therapeutic targets [33, 34].

TWIST1 is known as one of EMT-TFs that plays some critical roles in the tumor growth’s initiation, invasion, and metastasis [9]. In addition, it participates in carcinoma progression that is associated with worse survival in various cancers [35] such as ovarian [36], bladder [37], liver [38], and kidney cancers [39]. Recently, special attention has been paid to TWIST1, because it contributes in the generations of CSCs and vasculogenic mimicry (VM) through inducing stemness properties and endothelial markers [15, 40]. The induction of EMT, as well as the phenotype of the CSC by TWIST1, enhances migration and invasion in CRC [40]. CSCs are known as subpopulations of cancer cells that are responsible for tumor progression, metastasis, angiogenesis, and resistance to chemotherapeutic agents [41, 42].

In the present study, we investigated the TWIST1 expression in a large sample of CRC patients by considering scoring as well as separately analyzing the nuclear and cytoplasmic TWIST1 expressions. Although most of the previous results have demonstrated cytoplasmic TWIST1 expression, very few studies reported nuclear TWIST1 expression in CRC [43–45]. Nuclear and cytoplasmic TWIST1 expressions were observed in all CRC samples of this study. Correspondingly, this result confirms earlier findings reported that most tumor cells use EMT-TFs [9]. As expected, our results demonstrate that CRC tissues had higher cytoplasmic and nuclear TWIST1 expression’s levels compared to adjacent normal tissues, which is consistent with previous results reporting cytoplasmic TWIST1 expression in CRC and normal tissues [46]. Our investigation on the increased nuclear TWIST1 expression levels showed a statistically significant association with distant metastasis among CRC patients, while no significant association was found between the cytoplasmic expression of TWIST1 and distant metastasis. Accordingly, this result is in line with the claim that cytoplasm translocation to the nucleus of TWIST1 is associated with late events in CRC [43]. The significance of TWIST1 expression in CRC is still controversial, in a way that some studies have shown that expression level of TWIST1 is positively correlated with lymph node metastasis and stage among CRC cases [14, 46]. However, the other CRC studies have reported no relationship among TWIST1 expression and metastasis and stage [47, 48]. These differences can be explained, in part, by considering the fact that TWIST1 acts as EMT-TF in the nucleus [9, 49] and these studies have not distinguished subcellular location of TWIST1 expression or they have only focused on cytoplasmic TWIST1 expression. To the best of our knowledge, TWIST1 contributes to cancer progression by nucleosome remodeling that alters the regulation of some other factors such as the reduced E-cadherin and induced BMI1 proto-oncogene leading to the increased motility of tumor cells and cancer stemness features, respectively [11, 49].

Although a few studies in the past reported nuclear expression of TWIST1 in CRC tissues [43–45], we showed for the first time in the current study a statistically significant association between nuclear expression of TWIST1 and survival outcomes in CRC patients. Our findings showed that CRC patients with a higher expression of nuclear TWIST1 had statistically significant lower DSS and PFS rates compared to those with a lower expression of nuclear TWIST1. Moreover, CRC patients who showed a higher level of TWIST1 had shorter 5-year survival rate for DSS and PFS compared to those with a low expression. Furthermore, we found that tumor differentiation is an independent prognostic factor for DSS and PFS in nuclear expression pattern, while there was no association among TWIST1 expression and clinicopathological features and survival outcomes in cytoplasmic expression. In this study, we showed that TWIST1 protein expression is a prognostic marker for DSS and PFS in CRC patients (as found in univariate analysis). However, this association wasn’t independently significant in the multivariate analysis. As there was a positive trend in this association, increasing the number of the cases may have improved this value in multivariate analysis. These results suggest that nuclear TWIST1 expression might be considered as a predictor for the poor survival in CRC patients. Moreover, our result highlights the importance of nuclear expression compared to the cytoplasmic expression of TWIST1, because the nucleus is known as a site for the initiation of cell reprogramming by EMT-TFs. It was found that TWIST1 mostly depends on the activation of the TGF-β receptor for chromatin binding that induce migration and invasion [50].

In the current study, we have also investigated CD105 expression, as an accessory for TGF-β receptor, in endothelial and tumor cells of CRC tissues that plays a role in the TGF-β signaling pathway. TGF-β signaling can activate EMT leading to the induced CSC formation in the epithelial cells [51, 52]. Although the role of TGF-β receptor was indicated in EMT, few reports have demonstrated that CD105 is involved in EMT [20, 21]. Moreover, CD105 is recognized as a more appropriate marker for angiogenesis that is associated with poor prognosis in CRC [53, 54]. In addition, our finding exhibited that endothelial CD105 expression was higher in CRC tissues compared to adjacent normal tissues, which is in line with the results of a study by *Zeljko* et al. [55]. Besides, we observed and noticed of CD105 expression in tumor cells, while very few studies have reported cytoplasmic CD105 in tumor cells of CRC patients [27]. Data analysis also showed a statistically significant association between cytoplasmic of CD105 expressions of tumor cells and TNM stage in CRC patients. Stage IV, as the most advanced stage that occurred distant metastasis, was a higher level of tumoral cytoplasmic CD105 expression in comparison with a lower stage II that showed the association of cytoplasmic CD105 expression in tumor cells with tumor aggressiveness in CRC. In a recent study, cytoplasmic expression of CD105 in tumor cells was observed in the samples of less aggressive colon cancer [27], while CD105 expression in tumor cells was correlated with the advanced tumor stage of kidney and ovarian cancers [25, 56]. We have also observed a positive significant correlation between CD105 expression in two different cells (endothelial cells and cytoplasm of tumor cells) in CRC tissues. Accordingly, this result may indicate that both types of cells use CD105 expression with the same function in invasion and metastasis events. Unlike other studies performed in this area, we found no significant association between CD105 expression and its survival in CRC patients. In the current study, the follow-up data was available for 138 out of 223 CRC patients in TWIST1 and 121 out of 208 CRC patients in CD105. If we could obtain the information about the patient’s survival outcomes and therefore increased the number of cancer-related deaths or events, the survival analysis might get significant for CD105 and prognostic value of TWIST1 and CD105 expressions may be more accurately estimated. In fact, by accessing all survival data, prognostic value of TWIST1 and CD105 expression may be increased.

As far as we know, the function of endothelial CD105 expression is largely known in CRC [14, 45]; however, the role of tumoral cytoplasmic expression of CD105 is still unclear. Studies on renal cancer introduced CD105 marker in tumor cells as a cancer stem cell marker (CSC) [20, 57, 58] that can affect EMT [20] and contribute to chemoresistance in renal CSCs [58]. A study performed on human hepatocellular carcinoma cells (HCCs) suggested that CD105 is not only an endothelial cell marker, but it is also expressed in liver CSCs with mesenchymal cell features [21]. Moreover, CD105 expression was shown to be related to CSC properties in ovarian cancer cells associated with poor prognosis and distant metastasis [56].

## Conclusions

Overexpression of TWIST1 and CD105 was detected in CRC tissues compared to the adjacent normal tissue samples. Our findings for the first time revealed that the increased nuclear expression of TWIST1, rather than its cytoplasmic expression, is associated with more advanced diseases as well as poor survival outcomes in CRC patients in univariate analysis. In addition, we showed that tumor differentiation was an independent prognostic variable for PFS and DSS in CRC patients. According to our findings, we suggest to pay more attention to the role of TWIST1 expression site in cancer cell’s function and progression, because TWIST1 plays a critical role for the initiation of cell reprogramming in the nucleus that is known as an important factor for EMT induction and generation, as well as the maintenance of CSCs. The present study indicated cytoplasmic and endothelial CD105 expressions in CRC. Moreover, our results highlight the cytoplasmic expression of CD105 in tumor cells for the prediction of progression and aggressive behavior in CRC. In this regard, accesses to all the information about the patient’s survival outcomes are required to improve our knowledge on CD105, to clarify the prognostic impact of CD105 protein expression on patients with CRC.

## Data Availability

The analyzed data during the current study are available from the corresponding author on reasonable request.

## References

[1] Bray F, Ferlay J, Soerjomataram I, Siegel RL, Torre LA, Jemal A (2018). Global cancer statistics 2018: GLOBOCAN estimates of incidence and mortality worldwide for 36 cancers in 185 countries. CA Cancer J Clin.

[2] Brenner H, Kloor M, Pox CP (2014). Colorectal cancer. Lancet..

[3] Peluso G, Incollingo P, Calogero A, Tammaro V, Rupealta N, Chiacchio G, et al. Current tissue molecular markers in colorectal Cancer: a literature review. Biomed Res Int. 2017:2605628. 10.1155/2017/2605628 PMC5682052 29214162

[4] Zhou P, Li B, Liu F, Zhang M, Wang Q, Liu Y, Yao Y, Li D (2017). The epithelial to mesenchymal transition (EMT) and cancer stem cells: implication for treatment resistance in pancreatic cancer. Mol Cancer.

[5] Guarino M (2007). Epithelial-mesenchymal transition and tumour invasion. Int J Biochem Cell Biol.

[6] Lamouille S, Xu J, Derynck R (2014). Molecular mechanisms of epithelial-mesenchymal transition. Nat Rev Mol Cell Biol.

[7] Ieda T, Tazawa H, Okabayashi H, Yano S, Shigeyasu K, Kuroda S, Ohara T, Noma K, Kishimoto H, Nishizaki M, Kagawa S, Shirakawa Y, Saitou T, Imamura T, Fujiwara T (2019). Visualization of epithelial-mesenchymal transition in an inflammatory microenvironment-colorectal cancer network. Sci Rep.

[8] Gurzu S, Silveanu C, Fetyko A, Butiurca V, Kovacs Z, Jung I (2016). Systematic review of the old and new concepts in the epithelial-mesenchymal transition of colorectal cancer. World J Gastroenterol.

[9] Brabletz T, Kalluri R, Nieto MA, Weinberg RA (2018). EMT in cancer. Nat Rev Cancer.

[10] Piccinin S, Tonin E, Sessa S, Demontis S, Rossi S, Pecciarini L, Zanatta L, Pivetta F, Grizzo A, Sonego M, Rosano C, Tos APD, Doglioni C, Maestro R (2012). A "twist box" code of p53 inactivation: twist box: p53 interaction promotes p53 degradation. Cancer Cell.

[11] Zhao Z, Rahman MA, Chen ZG, Shin DM (2017). Multiple biological functions of Twist1 in various cancers. Oncotarget..

[12] Zhu QQ, Ma C, Wang Q, Song Y, Lv T (2016). The role of TWIST1 in epithelial-mesenchymal transition and cancers. Tumour Biol.

[13] Singh S, Mak IWY, Handa D, Ghert M. The role of TWIST in angiogenesis and cell migration in Giant cell tumor of bone. Adv Biol. 2014:1–8.

[14] Zhu DJ, Chen XW, Zhang WJ, Wang JZ, Ouyang MZ, Zhong Q, Liu CC (2015). Twist1 is a potential prognostic marker for colorectal cancer and associated with chemoresistance. Am J Cancer Res.

[15] Chen H-F, Huang C-H, Liu C-J, Hung J-J, Hsu C-C, Teng S-C, Wu KJ (2014). Twist1 induces endothelial differentiation of tumour cells through the Jagged1-KLF4 axis. Nat Commun.

[16] Sun T, Zhao N, Zhao XL, Gu Q, Zhang SW, Che N, Wang XH, du J, Liu YX, Sun BC (2010). Expression and functional significance of Twist1 in hepatocellular carcinoma: its role in vasculogenic mimicry. Hepatology..

[17] Hao Y, Baker D, Ten Dijke P (2019). TGF-beta-Mediated Epithelial-Mesenchymal Transition and Cancer Metastasis. Int J Mol Sci.

[18] Xu J, Lamouille S, Derynck R (2009). TGF-beta-induced epithelial to mesenchymal transition. Cell Res.

[19] Cheifetz S, Bellon T, Cales C, Vera S, Bernabeu C, Massague J (1992). Endoglin is a component of the transforming growth factor-beta receptor system in human endothelial cells. J Biol Chem.

[20] Hu J, Guan W, Yan L, Ye Z, Wu L, Xu H. Cancer stem cell marker Endoglin (CD105) induces epithelial Mesenchymal transition (EMT) but not metastasis in clear cell renal cell carcinoma. Stem Cells Int. 2019:9060152. 10.1155/2019/9060152 PMC6444238 31015843

[21] Nomura Y, Yamashita T, Oishi N, Nio K, Hayashi T, Yoshida M, Hayashi T, Hashiba T, Asahina Y, Okada H, Sunagozaka H, Takatori H, Honda M, Kaneko S (2017). De novo emergence of Mesenchymal stem-like CD105(+) Cancer cells by cytotoxic agents in human hepatocellular carcinoma. Transl Oncol.

[22] Nassiri F, Cusimano MD, Scheithauer BW, Rotondo F, Fazio A, Yousef GM, Syro LV, Kovacs K, Lloyd RV (2011). Endoglin (CD105): a review of its role in angiogenesis and tumor diagnosis, progression and therapy. Anticancer Res.

[23] Bernabeu C, Lopez-Novoa JM, Quintanilla M (2009). The emerging role of TGF-beta superfamily coreceptors in cancer. Biochim Biophys Acta.

[24] Minhajat R, Mori D, Yamasaki F, Sugita Y, Satoh T, Tokunaga O (2006). Organ-specific endoglin (CD105) expression in the angiogenesis of human cancers. Pathol Int.

[25] Saroufim A, Messai Y, Hasmim M, Rioux N, Iacovelli R, Verhoest G, Bensalah K, Patard JJ, Albiges L, Azzarone B, Escudier B, Chouaib S (2014). Tumoral CD105 is a novel independent prognostic marker for prognosis in clear-cell renal cell carcinoma. Br J Cancer.

[26] Nikiteas NI, Tzanakis N, Theodoropoulos G, Atsaves V, Christoni Z, Karakitsos P, Lazaris AC, Papachristodoulou A, Klonaris C, Gazouli M (2007). Vascular endothelial growth factor and endoglin (CD-105) in gastric cancer. Gastric Cancer.

[27] Nogues A, Gallardo-Vara E, Zafra MP, Mate P, Marijuan JL, Alonso A (2020). Endoglin (CD105) and VEGF as potential angiogenic and dissemination markers for colorectal cancer. World J Surg Oncol.

[28] Romani AA, Borghetti AF, Del Rio P, Sianesi M, Soliani P (2006). The risk of developing metastatic disease in colorectal cancer is related to CD105-positive vessel count. J Surg Oncol.

[29] Weiser MR (2018). AJCC 8th edition: colorectal Cancer. Ann Surg Oncol.

[30] Erfani E, Roudi R, Rakhshan A, Sabet MN, Shariftabrizi A, Madjd Z (2016). Comparative expression analysis of putative cancer stem cell markers CD44 and ALDH1A1 in various skin cancer subtypes. Int J Biol Markers.

[31] Camp RL, Charette LA, Rimm DL (2000). Validation of tissue microarray technology in breast carcinoma. Lab Investig.

[32] McCarty KS, Szabo E, Flowers JL, Cox EB, Leight GS, Miller L (1986). Use of a monoclonal anti-estrogen receptor antibody in the immunohistochemical evaluation of human tumors. Cancer Res.

[33] Thiery JP, Acloque H, Huang RY, Nieto MA (2009). Epithelial-mesenchymal transitions in development and disease. Cell..

[34] Georgakopoulos-Soares I, Chartoumpekis DV, Kyriazopoulou V, Zaravinos A (2020). EMT factors and metabolic pathways in Cancer. Front Oncol.

[35] Wushou A, Hou J, Zhao YJ, Shao ZM (2014). Twist-1 up-regulation in carcinoma correlates to poor survival. Int J Mol Sci.

[36] Hosono S, Kajiyama H, Terauchi M, Shibata K, Ino K, Nawa A, Kikkawa F (2007). Expression of twist increases the risk for recurrence and for poor survival in epithelial ovarian carcinoma patients. Br J Cancer.

[37] Fondrevelle ME, Kantelip B, Reiter RE, Chopin DK, Thiery JP, Monnien F, Bittard H, Wallerand H (2009). The expression of twist has an impact on survival in human bladder cancer and is influenced by the smoking status. Urol Oncol.

[38] Niu RF, Zhang L, Xi GM, Wei XY, Yang Y, Shi YR, Hao XS (2007). Up-regulation of twist induces angiogenesis and correlates with metastasis in hepatocellular carcinoma. J Exp Clin Cancer Res.

[39] Rasti A, Madjd Z, Abolhasani M, Mehrazma M, Janani L, Saeednejad Zanjani L, Asgari M (2018). Cytoplasmic expression of Twist1, an EMT-related transcription factor, is associated with higher grades renal cell carcinomas and worse progression-free survival in clear cell renal cell carcinoma. Clin Exp Med.

[40] Yang Y, Wang G, Zhu D, Huang Y, Luo Y, Su P, Chen X, Wang Q (2017). Epithelial-mesenchymal transition and cancer stem cell-like phenotype induced by Twist1 contribute to acquired resistance to irinotecan in colon cancer. Int J Oncol.

[41] Nguyen LV, Vanner R, Dirks P, Eaves CJ (2012). Cancer stem cells: an evolving concept. Nat Rev Cancer.

[42] Ayob AZ, Ramasamy TS (2018). Cancer stem cells as key drivers of tumour progression. J Biomed Sci.

[43] Mohammed AS, Kandil MA, Asaad NY, Aiad HA, El Tahmoudy MA, Hemida AS. Immunohistochemical expression of Twist in colorectal carcinoma. Menoufia Med J. 2015;28(3):725–33. 10.4103/1110-2098.165816.

[44] Hong R, Lim S-C (2009). Overexpression of twist in colorectal adenocarcinoma. Basic Appl Pathol.

[45] Celesti G, Di Caro G, Bianchi P, Grizzi F, Basso G, Marchesi F (2013). Presence of Twist1-positive neoplastic cells in the stroma of chromosome-unstable colorectal tumors. Gastroenterology..

[46] Yusup A, Huji B, Fang C, Wang F, Dadihan T, Wang HJ, Upur H (2017). Expression of trefoil factors and TWIST1 in colorectal cancer and their correlation with metastatic potential and prognosis. World J Gastroenterol.

[47] Kim YH, Kim G, Kwon CI, Kim JW, Park PW, Hahm KB (2014). TWIST1 and SNAI1 as markers of poor prognosis in human colorectal cancer are associated with the expression of ALDH1 and TGF-beta1. Oncol Rep.

[48] Gomez I, Pena C, Herrera M, Munoz C, Larriba MJ, Garcia V (2011). TWIST1 is expressed in colorectal carcinomas and predicts patient survival. PLoS One.

[49] Qin Q, Xu Y, He T, Qin C, Xu J (2012). Normal and disease-related biological functions of Twist1 and underlying molecular mechanisms. Cell Res.

[50] Dragoi D, Krattenmacher A, Mishra VK, Schmidt JM, Kloos UJ, Meixner LK, Hauck SM, Buggenthin F, Schwartz D, Marr C, Johnsen SA, Scheel CH (2016). Twist1 induces distinct cell states depending on TGFBR1-activation. Oncotarget..

[51] Liu S, Chen S, Zeng J (2018). TGFbeta signaling: a complex role in tumorigenesis (review). Mol Med Rep.

[52] Lichner Z, Saleh C, Subramaniam V, Seivwright A, Prud'homme GJ, Yousef GM (2015). miR-17 inhibition enhances the formation of kidney cancer spheres with stem cell/ tumor initiating cell properties. Oncotarget..

[53] Jung I, Gurzu S, Raica M, Cimpean AM, Szentirmay Z (2009). The differences between the endothelial area marked with CD31 and CD105 in colorectal carcinomas by computer-assisted morphometrical analysis. Romanian J Morphol Embryol.

[54] Dallas NA, Samuel S, Xia L, Fan F, Gray MJ, Lim SJ, Ellis LM (2008). Endoglin (CD105): a marker of tumor vasculature and potential target for therapy. Clin Cancer Res.

[55] Martinovic Z, Kovac D, Martinovic M. Prognostic significance of microvessel density determining by Endoglin in stage II rectal carcinoma: a retrospective analysis. Gastroenterol Res Pract. 2015:504179. 10.1155/2015/504179 PMC4454763 26089870

[56] Zhang J, Yuan B, Zhang H, Li H (2019). Human epithelial ovarian cancer cells expressing CD105, CD44 and CD106 surface markers exhibit increased invasive capacity and drug resistance. Oncol Lett.

[57] Bussolati B, Bruno S, Grange C, Ferrando U, Camussi G (2008). Identification of a tumor-initiating stem cell population in human renal carcinomas. FASEB J.

[58] Hu J, Guan W, Liu P, Dai J, Tang K, Xiao H, Qian Y, Sharrow AC, Ye Z, Wu L, Xu H (2017). Endoglin is essential for the maintenance of self-renewal and Chemoresistance in renal Cancer stem cells. Stem Cell Rep.

